# Establishment and Characterization of a Long-Term Ovarian Cell Line (SBO) from Asian Seabass (*Lates calcarifer*) Expressing Germline Stem Cell Markers

**DOI:** 10.3390/ijms27104608

**Published:** 2026-05-21

**Authors:** Ruobing Zhang, Zeyu Zhan, Minglian Zhao, Yiying Li, Hongyan Xu

**Affiliations:** Key Laboratory of Fresh-Water Fish Reproduction and Development Ministry of Education, Key Laboratory of Aquatic Sciences of Chongqing, Integrative Science Center of Germplasm Creation in Western China (CHONGQING) Science City & College of Fisheries, Southwest University, Chongqing 402460, China; 15638740801@163.com (R.Z.); swu2023000@163.com (Z.Z.); z2938966366@163.com (M.Z.); 15213336661@163.com (Y.L.)

**Keywords:** germline stem cells, *Lates calcarifer*, SBO, female germline stem cell

## Abstract

Germline stem cells (GSCs) are crucial for gametogenesis, genetic conservation, and molecular breeding. Although GSCs lines have been well studied in mammals and several model teleost species, progress in commercial marine teleosts remains limited. In this study, we report a successful establishment of a long-term stable ovarian cell line derived from the Asian seabass (*Lates calcarifer*), designated the Seabass Ovarian (SBO) cell line. Ovaries were dissociated using a combined collagenase–trypsin digestion protocol; the cells were propagated and maintained in DMEM supplemented with bFGF, LIF, and fish serum. The SBO cells exhibited strong alkaline phosphatase activity. Furthermore, the cultured cells robustly expressed both germ-cell specific markers (Vasa) and pluripotency associated proteins (Nanog, SSEA-1). These findings indicate the successful isolation and long-term maintenance of an ovarian cell line expressing female germline stem cell markers from Asian seabass ovaries. The established cell line not only provides a valuable in vitro model for elucidating the mechanisms behind germ cell differentiation but also serves as a crucial cellular resource for advancing genetic breeding, germplasm preservation, and surrogate broodstock technologies in marine teleosts.

## 1. Introduction

Asian seabass, also known as barramundi, is a euryhaline demersal fish widely distributed throughout the Indo-West Pacific region [1]. Characterized by extremely rapid growth rate, robust tolerance to high-density farming environments, and excellent flesh quality, the Asian seabass has emerged as one of the most economically valuable marine aquaculture species globally in tropical and subtropical regions [2]. As a typical protandrous hermaphroditic fish, in which individuals usually mature as a male, and later, at a certain age or body size, naturally undergo sex reversal to become a female [3,4], the Asian seabass is considered as a unique biological model for studying sex determination, gonadal development, and endocrine regulation in non-model marine teleosts [5]. However, to date, studies on the mechanisms of oogenesis and the sex-reversal networks in Asian seabass has relied overwhelmingly on in vivo observations and histological analyses. The lack of a stably proliferating in vitro ovarian cell model has hindered the functional verification of reproduction-related target genes and cellular-level manipulations.

Germline stem cells (GSCs) represent the only adult stem cell population in vertebrates capable of transmitting genetic information to the offspring and ensuring the continuity of a species [6]. For over half a century, the dogma that “female mammals are born with a fixed and non-renewable reserve of oocytes” served as the fundamental paradigm of reproductive biology [7]. However, this traditional concept was challenged when Bukovsky et al. provided evidence of oocyte renewal and germ cell formation in adult human and rat ovaries [8]. Subsequently, Zou et al. established a long-term in vitro culture system for mouse fGSCs and demonstrated that these cells could produce normal offspring through transplantation [9]. In recent years, the successful isolation and characterization of ovarian stem cells in humans and other mammals have further confirmed the ubiquitous presence of fGSCs in adult animal ovaries [10]. These in vitro cultured germline stem cells not only provide an unprecedented platform for dissecting the molecular mechanisms of oogenesis but also pave new way for fertility preservation in reproductive medicine and genetic breeding of superior fish species.

Following the rapid advancements in mammalian fGSC research, progress has also been achieved in the in vitro culture of germ cells in aquatic animals, particularly fishes and reptiles. For instance, ovarian stem cells were successfully isolated and cultured from the Chinese soft-shell turtle (*Pelodiscus sinensis*) [11] and the Asian Yellow Pond turtle (*Mauremys mutica*) [12]. In teleosts, germline stem cells from model species such as zebrafish (*Danio rerio*) [13] and medaka [14] (*Oryzias latipes*) have been extensively investigated. Based on the successful isolation and identification of fish GSCs, Yoshizaki’s team pioneered the technology of “surrogate broodstock”, the donor spermatogonial or oogonial stem cells transplanted into recipient larvae can be integrated into the genita ridge of recipient larvae, thus leading to the production of donor-derived gametes [15]. Furthermore, combined with cryopreservation technologies, in vitro cultured GSCs have become the core resources for establishing germplasm banks for conserving and restoring the endangered fish species [16].

Although the establishment and application of germline stem cell lines are well developed in freshwater model fishes and certain reptiles, overcoming the technical bottlenecks to establish germ cell lines (especially ovarian cell lines) for non-model marine commercial fishes remains a formidable challenge [17]. The in vitro culture requirements for marine fish cells—such as osmotic pressure, trophic factors, and attachment matrices—are notoriously stringent, providing valuable methodological insights for germ cell culture in sex-reversing marine teleosts. Nevertheless, to date, no long-term in vitro ovarian cell line or germline stem cell line has been reported for Asian seabass.

The marine fish cells are prone to rapid senescence, excessive proliferation of fibroblasts, or loss of stem cell characteristics during a long-term in vitro culture. Recently, Zhong et al. optimized culture conditions and successfully established a spermatogonial stem cell line with meiotic potential from the orange-spotted grouper (*Epinephelus coioides*, a protogynous hermaphroditic fish) [18], providing invaluable insights for the in vitro culture of germ cells in sex-reversing marine teleosts. Nevertheless, there is no report on the long-term stable ovarian cell line or germline stem cell line derived from Asian seabass.

In this study, we aimed to establish and characterize a stable ovarian cell line derived from Asian seabass. By optimizing an in vitro culture system enriched with various growth factors, we successfully isolated and long-term subcultured Asian seabass ovarian cells (designated as the SBO cell line). We systematically evaluated the biological characteristics of this cell line through the morphological observations, chromosome karyotype analysis, and alkaline phosphatase staining and comprehensively characterized its stemness markers (e.g., Nanog, Vasa, PCNA) using qPCR, immunofluorescence, and Western blot. We conducted small-molecule inhibitor assays (e.g., A83-01, CHIR99021, Y-27632) to assess genetic stability [19] and optimize culture conditions. The SBO cell line established in this study not only fills the critical gap in reproductive biology of fish species but also serves as an excellent in vitro platform for unraveling the oogenesis mechanisms in marine sex-reversing fishes, in vitro gametogenesis, efficient functional gene editing, and the long-term cryopreservation of elite marine germplasm resources.

## 2. Results

### 2.1. Establishment of Ovarian Cells Line

**Figure 1:**
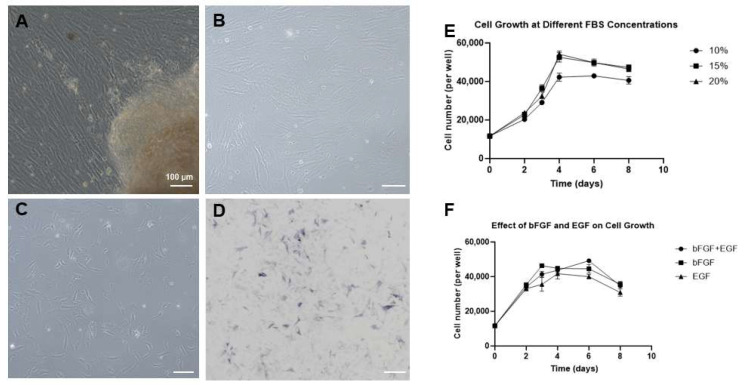
Morphological observation, alkaline phosphatase activity, and growth characteristics of the Asian seabass ovarian cell line (SBO). The cells were observed under an inverted microscope. (**A**) Primary culture of ovarian cells, showing cells growing outward from the edges of tissue blocks. (**B**) Morphological characteristics of SBO cells in monolayer at passage 15 (p15). (**C**) SBO cells at passage 35 (p35), maintaining a uniform and stable morphology. (**D**) Assessment of alkaline phosphatase (AP) activity in SBO cells at passage 20. Most of the cells exhibited prominent positive staining (blue–purple), a typical feature of stem cells. (**E**) Growth curves of p20 SBO cells under different fetal bovine serum (FBS) concentrations (10%, 15%, 20%). (**F**) Growth curves of p20 SBO cells cultured with different combinations of growth factors (bFGF + EGF, bFGF alone, EGF alone). Scale bars, 100 µm.

In the primary culture of Asian seabass ovarian cells, a heterogeneous population of cells with diverse morphologies was observed migrating and growing out from the edges of the tissue blocks (Figure 1A). These mainly included epithelial-like, fibroblast-like, and a few round-shaped cells. In the initial passages, the cells grew relatively slowly. However, after several passages of subculture, the fibroblast-like and epithelial-like populations gradually became dominant, and the cells became more proliferative and grew faster. After continuous in vitro subculture to the 15th passage, the cells reached confluence to form a monolayer, and their morphology became more uniform (Figure 1B). As the culture progressed to the 35th passage, the cells still exhibited high proliferation capacity and stably grew in a monolayer (Figure 1C), with consistent morphological characteristics. These observations indicate that a stable ovarian-derived cell line from Asian seabass, designated as SBO, has been successfully established in vitro under the optimized culture conditions.

Alkaline phosphatase (AP) activity is a classical biochemical indicator used to verify the stem cell characteristics of in vitro cultured cells. To primarily evaluate the stemness properties of SBO cells, we performed AP staining analysis on cells at the 20th passage. The staining results demonstrated that the vast majority of the in vitro cultured SBO cells exhibited prominent positive staining (blue–purple) (Figure 1D), a typical feature of stem cells. This indicates that the in vitro long-term cultured SBO cells possess a high level of alkaline phosphatase activity.

To investigate the optimal in vitro growth conditions for SBO cells, we evaluated the effects of different fetal bovine serum (FBS) concentrations and combinations of growth factors on the proliferation of p20 SBO cells. In the FBS concentration gradient experiment, SBO cells grew normally under 10%, 15%, and 20% FBS concentrations, showing similar proliferation trends. However, the proliferation rate and peak cell number in the 15% and 20% FBS groups were significantly higher than those in the 10% group, with no obvious difference between the 15% and 20% groups. This suggests that 15% FBS concentration is sufficient to meet the optimal growth requirements of SBO cells (Figure 1E). In the growth factor optimization experiment, groups containing bFGF (alone or in combination with EGF) exhibited a stronger proliferation-promoting effect in the early stage of culture, whereas the growth-promoting effect of EGF alone was relatively weaker (Figure 1F). Based on the analysis of these growth curves, the optimal nutritional and factor supplementation conditions for the in vitro culture of SBO cells were established.

### 2.2. Selected Marker Genes Examined in the SBO Cells

**Figure 2:**
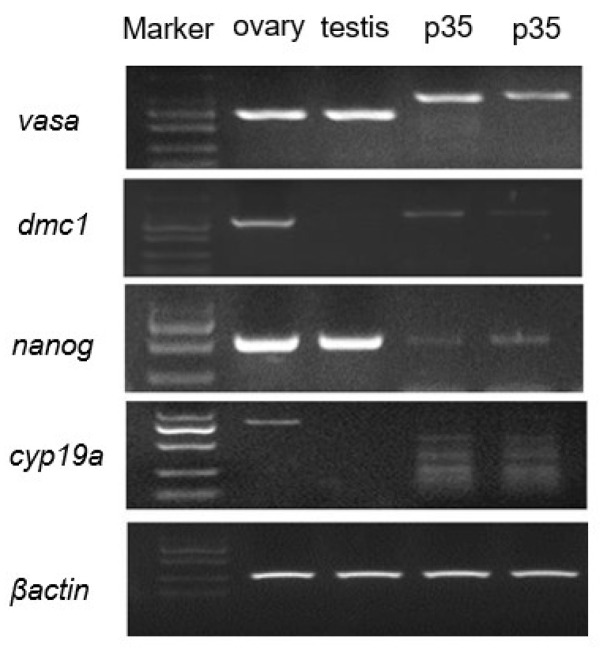
Expression of marker genes in the SBO cells. The mRNA expressions of related markers were examined in SBO cells at passage 35. The cDNA of Asian seabass ovary, testis, and SBO cells was used as templates for reverse transcription polymerase chain reaction (RT-PCR). SBO cells exhibited robust expression of germ cell markers (Vasa) and moderate expression of dmc1 and cyp19a. Moderate expression of the key stem cell marker Nanog. The housekeeping gene β-actin was used as an internal control.

To further verify the germline and stem cell characteristics of the SBO cells, we examined the mRNA expression profiles of several well-known cell markers in SBO cells at passage 35 using RT-PCR (Figure 2). The results showed that the SBO cells exhibited robust expression of the germ cell marker Vasa, and moderate expression of dmc1 and cyp19a. Moderate expression of the key stem cell marker Nanog was also observed. The housekeeping gene β-actin was used as an internal control. This specific gene expression profile further substantiates the molecular characteristics of the in vitro long-term cultured SBO cells.

**Figure 3:**
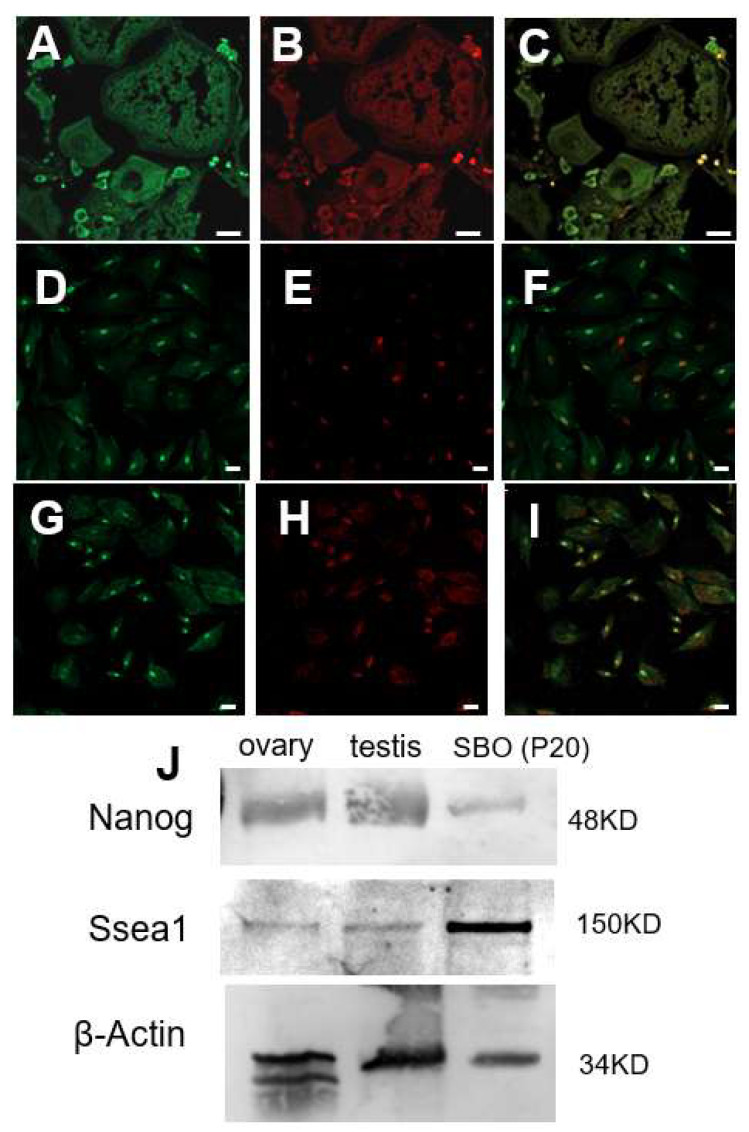
Expression and cellular localization analysis of Nanog and PCNA proteins in Asian seabass ovary tissue and SBO cells. (**A**–**C**) Immunofluorescence staining of the stem cell pluripotency marker Nanog on Asian seabass ovary tissue sections. (**A**) Green fluorescent signal of the Nanog protein, (**B**) red fluorescent signal of nuclei counterstained with propidium iodide (PI), and (**C**) the merged image. (**D**–**F**) Immunofluorescence staining of the Nanog protein in cultured SBO cells, showing predominant expression in the nuclei. (**D**) Nanog green fluorescence, (**E**) PI nuclear staining, and (**F**) the merged image. (**G**–**I**) Immunofluorescence staining of the proliferating cell nuclear antigen (PCNA) in the cultured SBO cells, exhibiting strong positive nuclear expression. (**G**) PCNA green fluorescence, (**H**) PI nuclear staining, and (**I**) the merged image. (**J**) Western blot analysis of stemness-related marker proteins Nanog and SSEA-1 in Asian seabass ovary tissue, testis tissue, and SBO cells at passage 20, with β-actin serving as the internal loading control. Scale bars, 50 µm.

To confirm the expression and localization patterns of stem cell markers in vivo at the protein level, we performed fluorescence immunostaining analysis of the Nanog protein on Asian seabass ovary tissue sections (Figure 3). As shown in Figure 3A, prominent green fluorescent signals were observed in specific germ cell populations within the ovary sections after labeling with a monoclonal anti-Nanog antibody. Meanwhile, propidium iodide (PI) was used to counterstain the nuclei in red (Figure 3B). In the merged images (Figure 3C), it can be clearly observed that the Nanog protein is specifically expressed in the germline stem cells/early oocytes of the Asian seabass ovary. This in vivo histological evidence not only establishes the reliability of Nanog as a valid marker for Asian seabass germline stem cells but also provides an important reference for the subsequent molecular characterization of the in vitro cultured SBO cell line.

To further verify the stem cell characteristics and proliferative activity of the SBO cells at the in vitro cultured cell level, we performed fluorescence immunostaining analysis of specific marker proteins on SBO cells at different passages (Figure 3D–I). As shown in Figure 3D–F, in the SBO cells at passage 34, the key pluripotency stem cell marker Nanog (green fluorescence) was predominantly localized within the nuclei, which is highly consistent with the expression pattern observed in the germ cells of the in vivo ovary tissue sections. Furthermore, we conducted immunofluorescence staining for the proliferating cell nuclear antigen (PCNA) on SBO cells at passage 41 (Figure 3G–I). Combined with the propidium iodide (PI) nuclear counterstaining (red), it can be clearly observed that the PCNA protein also exhibits strong positive expression localized in the nuclei of the SBO cells. This indicates that the SBO cells maintain a vigorous capacity for cell division and proliferation even after long-term in vitro subculturing. This protein expression evidence at the in vitro cellular level further corroborates the validity and stability of the SBO cell line as an in vitro cell model expressing germline stem cell markers for Asian seabass.

To further verify the stem cell characteristics of the SBO cells at the protein level, we evaluated the expression of stemness-related marker proteins in the ovary and testis tissues of Asian seabass, as well as in the 20th-passage SBO cells using Western blot analysis (Figure 3J). The results demonstrated that the stem cell pluripotency markers Nanog (48 KD) and SSEA-1 were both detectable in the Asian seabass ovary, testis, and the in vitro cultured SBO cells. Notably, these stemness markers exhibited distinct positive expression bands in the in vitro subcultured SBO cells, with the internal control protein β-actin (34 KD) stably expressed across all samples. These protein-level detection results are highly consistent with the aforementioned mRNA expression and immunofluorescence localization findings, providing further solid evidence that the in vitro long-term cultured SBO cells retain the typical characteristics of Asian seabass germline stem cells.

### 2.3. Effects of Different Small Molecule Inhibitors on the Proliferation of SBO Cells

**Figure 4:**
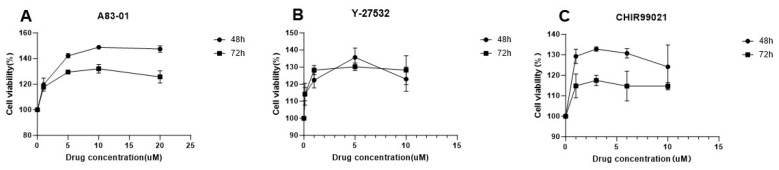
The effects of different inhibitors on SBO cells’ proliferation. The y-axis represents the relative cell viability to the control, and the x-axis represents the inhibitor concentration. (**A**) the cells incubated with different concentrations of A83-01 for 48 h and 72 h, respectively. (**B**) Cells with different concentrations of Y-27532 for 48 h and 72 h. (**C**) Cells with different concentrations of CHIR99021 for 48 h and 72 h.

To investigate the effects of different small molecule inhibitors on the growth of SBO cells, we treated the cells with A83-01, Y-27632, and CHIR99021 for 48 hours (48 h) and 72 hours (72 h), respectively, and assessed the changes in cell viability (Figure 4). As illustrated in Figure 4A, A83-01 significantly increased cell viability within the concentration range of 5–10 μM, peaking at nearly 150% at 48 h. The Y-27632 treatment group (Figure 4B) also showed an obvious proliferation-promoting effect at concentrations of 2.5–5 μM. Similarly, the cell viability in the CHIR99021 treatment group (Figure 4C) reached its highest point at approximately 5 μM. These results indicate that optimal concentrations of A83-01, Y-27632, and CHIR99021 can effectively maintain and promote the in vitro survival and proliferation of SBO cells, providing important concentration references for subsequent experiments involving the maintenance of cell stemness or directed differentiation using combinations of small molecules.

## 3. Discussion

Although the in vitro culture of germline stem cells (GSCs) has made significant progress in mammals [20] and some freshwater model fishes [21], the establishment of continuous germ cell lines for marine fishes—especially non-model commercial species with sex-reversing characteristics—remains a tremendous challenge [17,22]. Marine fish cells often have highly stringent requirements for osmotic balance, extracellular microenvironments, and nutritional support during in vitro culture and are always prone to early senescence, fibroblast overgrowth of tissues, or loss of cell proliferation potency during routine passaging. In the present study, we successfully isolated and established the first stable cell line (SBO) from Asian seabass ovaries. Through optimizing culture conditions and medium, the SBO cells have been stably subcultured for more than 35 passages in vitro, and cells still possess robust proliferation capacity. The result is comparable with the previously reported germline stem cell lines from freshwater model species such as zebrafish [23] and medaka [14], as well as marine hermaphroditic species like orange-spotted grouper [18].

The cultured SBO cells exhibited typical fibroblast-like and epithelial-like morphologies, similar to germline stem cells or ovarian cell lines previously reported in tomato grouper (*Cephalopholis sonnerati*) [24], half-smooth tongue sole (*Cynoglossus semilaevis*) [25], orange-spotted grouper [18], and Chinese soft-shell turtle [11]. The stable propagation of germline stem cells always depends on defined the culture medium and conditions. The cells’ growth curve assay showed that 15% FBS is sufficient to support SBO cells growth. Likewise, the combined supplementation of basic growth factor (bFGF) and epidermal growth factor (EGF) could promote the proliferation of SBO cells. Numerous previous studies have also demonstrated the effects of various exogenous growth factors on stem cells cultured in vitro. IGF2 maintains self-renewal and pluripotency of medaka embryonic stem cells by activating Erk1/2 and Akt signaling pathways [26]. bFGF is essential for maintaining the expression of spermatogenesis-related genes (*scp3* and *sox9a*) in marine medaka testicular germ cells [27]. bFGF also regulates the initiation and progression of spermatogenesis in medaka via somatic cells such as Sertoli cells [28].

Alkaline phosphatase activity is widely recognized as a hallmark of pluripotent and germline stem cells. The strong and widespread AP positivity observed in SBO cells provides initial biochemical evidence supporting their stem cell–like characteristics. In addition, the cultured SBO cells also expressed germline- and stemness-associated markers at both the transcript and protein levels, including Vasa, Nanog, and SSEA-1. Intriguingly, Vasa, a highly conserved germ cell marker across vertebrates [29]. Moreover, Nanog, as a core transcription factor maintaining cell pluripotency, acts synergistically with the endogenous core regulatory network to repress the expression of differentiation-related genes, thereby locking the cells in an undifferentiated ground state of self-renewal [30]. Meanwhile, SSEA-1 (stage-specific embryonic antigen-1) is a typical pluripotent carbohydrate epitope on the surface of early embryonic stem cells and primordial germ cells (PGCs) [31]. The co-expression of Nanog and SSEA-1 proves that SBO cells retain a high degree of “stemness” even after a long-term in vitro subculture.

The proliferation of cultured SBO cells can be effectively regulated by small-molecule inhibitors (A83-01, CHIR99021, and Y-27632) commonly used in stem cell biology [32]. These molecules are known to modulate key signaling pathways involved in stem cell maintenance, including TGF-β, Wnt/β-catenin, and Rho/ROCK signaling. TGFβ receptor inhibitor (A83-01), GSK-3 inhibitor (CHIR99021), and ROCK inhibitor (Y-27632) could promote the in vitro survival and proliferation of SBO cells. Previous studies have shown that CHIR99021, a classical activator of the Wnt/β-catenin pathway, can effectively inhibit stem cell differentiation and maintain their self-renewal ground state [33]; meanwhile, Y-27632 can significantly reduce apoptosis (anoikis) during cell dissociation and subculturing by inhibiting the Rho/ROCK pathway [34]. The combination of A83-01 and a MEK inhibitor can replace exogenous transcription factors and significantly enhance induction efficiency [35]. In our study, treatment with these inhibitors significantly enhanced cell viability within the defined concentration ranges. The positive responses of SBO cells to these compounds imply that the cells’ stemness-related signaling networks remain functionally responsive, providing valuable index for developing techniques to in vitro maintain pluripotency or induce directed differentiation of cultured fish cells in future.

## 4. Materials and Methods

### 4.1. Fish and Ethics

All experiments were conducted in accordance with the approval of the Animal Care and Ethics Committee of Southwest University (Chongqing, China) for this research (Approval Code: IACUC-20210120-01; Approval Date: 10 January 2021). All experimental protocols and methods were performed in accordance with the relevant guidelines and regulations. Adult Asian seabass (~5-year-old; body weight, ~5 kg) used in the experiment were collected from a fish farm in Haikou, Hainan. The fish were euthanized using 0.01% tricaine methanesulfonate (MS-222; Fisherbao, Hangzhou, China) until gill movement ceased and there was no response to mechanical stimulation. Then, they were sacrificed for tissue collection.

### 4.2. Isolation of Ovarian Cells

The dissected ovary tissues were washed three times with PBS supplemented with 1% penicillin-streptomycin (100 units/mL penicillin, 100 µg/mL streptomycin; Gibco, Grand Island, NY, USA). Subsequently, the tissues were minced into small pieces (approximately 1 mm^3^) and enzymatically digested with 1 mg/mL collagenase IV (Solarbio, Beijing, China) at 28 °C for 20 min, followed by treatment with 0.25% Trypsin-EDTA (Gibco, NY, USA) for 5 min to obtain a single-cell suspension. After centrifugation at 500× *g* for 5 min, the supernatant was removed. The resulting cell pellet was resuspended in complete culture medium (formulation given in Section 4.3) and seeded into 25 cm^2^ rectangular canted-neck cell culture flasks (Corning, New York, NY, USA).

### 4.3. Cell Culture

Isolated cells were cultured in the absence of feeder layers in culture flasks pre-coated with 0.1% gelatin (Macklin, Shanghai, China), following previously established protocols [14,36]. Since ESM medium has been demonstrated to support the long-term proliferation of medaka SG3, medaka embryonic stem cell lines [36,37], an ovarian stem cell line from soft-shell turtle [11], and an SSC line from orange-spotted grouper [18], it was used for the cultivation of Asian seabass ovarian (SBO) cells. The ESM medium formula was slightly modified as follows: Dulbecco’s Modified Eagle Medium (DMEM; Gibco, NY, USA) supplemented with 20 mM HEPES (Gibco), 15% fetal bovine serum (FBS; Gibco), 1% penicillin-streptomycin (Gibco), 10 ng/mL recombinant human bFGF (MedChemExpress, Monmouth Junction, NJ, USA), 10 ng/mL recombinant human LIF (MedChemExpress), 1 mM non-essential amino acids (Gibco), 1 mM sodium pyruvate (Gibco), 2 mM L glutamine (Gibco), 2 nM sodium selenite (Sigma-Aldrich, Burlington, MA, USA), 0.2% seabass serum, medaka embryo extract (equivalent to 2.5 embryos/mL), and 55 µM 2 mercaptoethanol (Gibco). The pH was adjusted to 7.5 using 10 M sodium hydroxide solution. Both ovarian tissues and isolated cells were maintained at 28 °C. Cells were subcultured at a split ratio of 1:3 using 0.25% trypsin-EDTA (Gibco) every 5–6 days during the initial passages and every 3–5 days after passage 10. Cell morphology was regularly monitored and imaged using an inverted microscope (Axio Observer 3; Zeiss, Oberkochen, Germany).

### 4.4. Alkaline Phosphatase Staining

Being propagated to approximately 70% confluence in a 12-well plate, cells were fixed with 4% paraformaldehyde (PFA; Biosharp, Hefei, China) for 10 min. After being washed twice with 0.2 M Tris-HCl solution (Beyotime, Shanghai, China), the cells were incubated in a substrate solution containing 0.188 mg/mL 5-bromo-4-chloro-3-indolyl phosphate (BCIP) and 0.375 mg/mL nitroblue tetrazolium chloride (NBT) (Roche, Basel, Switzerland) in the dark overnight at 4 °C. Subsequently, the cells were washed twice with PBS and imaged using an inverted microscope (Axio Observer 3; Zeiss, Oberkochen, Germany).

### 4.5. Western Blotting

Total protein was extracted from gonads and cultured cells using RIPA lysis buffer (Beyotime, Shanghai, China) supplemented with 1% Protease Inhibitor Cocktail (Roche, Basel, Switzerland). Equal amounts of protein (15 μg) were separated by 10% SDS-polyacrylamide gel electrophoresis (SDS-PAGE) and subsequently transferred onto PVDF membranes (Millipore, Billerica, MA, USA). After blocking with 5% non-fat milk or 2% bovine serum albumin (BSA; Sigma-Aldrich, Burlington, MA, USA) for 2 h at room temperature, the membranes were incubated overnight at 4 °C with the following primary antibodies: anti-Nanog (1:1000), anti-Ssea1 (1:1000), and anti-β-actin (1:1000; Merck, Darmstadt, Germany). Following three washes with TBS, the membranes were incubated with HRP-conjugated secondary antibodies (1:2000) for 1 h. Protein signals were visualized using an enhanced chemiluminescence (ECL) detection system (Tanon, Shanghai, China).

### 4.6. RT-PCR Analysis

Total RNA was extracted from dissected ovaries, testes, and cultured cells using TRIzol Reagent (Takara Bio, Kusatsu, Japan) according to the manufacturer’s instructions. Genomic DNA (gDNA) was removed by treatment with RNase-free DNase. The concentration and purity of the isolated RNA were quantified using a NanoQ™ microspectrophotometer (Implen, Munich, Germany), and RNA integrity was verified via 1% agarose gel electrophoresis. First-strand cDNA was synthesized from 1 μg of total RNA using the PrimeScript™ RT Reagent Kit with gDNA Eraser (Takara Bio). For the identification of marker genes, PCR amplification was performed using PrimeSTAR^®^ Max DNA Polymerase (Takara Bio). The PCR thermal cycling conditions consisted of an initial denaturation at 98 °C for 2 min, followed by 30 cycles of 98 °C for 10 s, 60 °C for 5 s (depending on the primer), and 72 °C for 5 s/kb, with a final extension at 72 °C for 5 min. The resulting PCR products were separated on a 1% agarose gel, visualized using a ChemiDoc™ XRS system (Bio-Rad, Hercules, CA, USA), and further validated by DNA sequencing. The β-actin gene was employed as an internal reference for normalization. Specific primer sequences used in this study are summarized in Table 1.

### 4.7. Fluorescent Immunostaining

To define cell types and developmental stages, histological analysis and immunostaining were conducted following established protocols [11,38,39]. Frozen ovarian sections (8 μm) and cultured cells were fixed with 4% paraformaldehyde for 10 min. After blocking with 5% BSA (BioFroxx, Einhausen, Germany) in PBS, samples were incubated overnight at 4 °C with primary antibodies: anti-Nanog (Monoclone antibody home-made, mouse, 1:500), anti SSEA-1 (Abcam, Cambridge, UK, mouse, 1:500), and anti–PCNA (Abcam, mouse, 1:500). The following day, samples were washed with PBST and blocked with 5% goat serum in PBS for 1 h before incubation with secondary antibodies for 1 h at room temperature. The HRP-conjugated anti-mouse IgG (Boster Bio, Wuhan, China) was diluted 1:2000 in PBS containing 2% goat serum. Signals were developed using the TSA™ Plus Fluorescence System (Akoya Biosciences, Marlborough, MA, USA). Cell nuclei were counterstained with 10 μg/mL DAPI. The sections and cells were imaged using a fluorescent microscope (Axio Observer 3; Zeiss, Oberkochen, Germany). Images were processed using ImageJ software (v1.8.0.112; NIH, Bethesda, MD, USA).

### 4.8. Optimization of Culture Medium

The effects of varying FBS concentrations and growth factors on cell proliferation were evaluated using the Enhanced CCK-8 kit (Biosharp, Anhui, China). Briefly, cells at passage 20 were seeded into 96-well plates (Corning, NY, USA) at 5000 cells/well in 100 μL of medium and incubated at 28 °C for 24 h. To determine optimal serum requirements, cells were cultured in media containing 10%, 15%, or 20% FBS. Simultaneously, to assess growth factor supplementation, cells were treated with 10 ng/mL bFGF, 20 ng/mL EGF, or a combination of both. Cell proliferation was monitored on days 2, 3, 4, 6, and 8 post-treatment. At each time point, 10 μL of CCK-8 reagent was added and incubated at 28 °C for 5 h in the dark. Absorbance was measured at 450 nm. Recorded values were converted into cell numbers based on a predetermined standard curve to generate growth kinetics plots. Data are presented as mean ± SD (n = 3).

### 4.9. Cell Viability Analysis

To investigate the impact of chemical induction on survival, SBO cell viability was measured after treatment with small-molecule inhibitors. Cells were seeded into 96-well plates at 3000 cells/well in triplicate and allowed to adhere overnight. Cells were then treated with the following: valproic acid (VPA; Merck, Darmstadt, Germany) at 0.25–2 μM; A83-01 (MedChemExpress, NJ, USA) at 1–20 μM; CHIR99021 (MedChemExpress) at 1–10 μM; and Y-27632 (Beyotime, Shanghai, China) at 0.1–10 μM. Untreated cells and medium-only wells served as controls. Viability was recorded at 48 h and 72 h using the CCK-8 kit (Biosharp). At each time point, 10 μL of CCK-8 solution was added and incubated at 28 °C for 5 h. Absorbance was measured at 450 nm, and relative viability was calculated by normalizing the absorbance of treated wells to that of the untreated controls after background subtraction.

## 5. Conclusions

In a summary, in this study an ovarian cell line, called as SBO, was successfully established in Asian seabass, providing a stable and well-characterized ovarian cell line. This cell line expressed germline stem cell markers and would be used for further investigations on reproductive biology of marine fish. This cell line also would be a powerful in vitro tool for studies in germ cell biology, functional gene analysis, surrogate broodstock technology, and long-term conservation of elite germplasm resources in marine fish species.

## Figures and Tables

**Table 1:** The specific primer sequences used in this study.

Primer Name	Primer Sequence
Sb-vasa F1	GAGCCTGAGGCCATCATTGT
Sb-vasa R1 Sb-dmc1 F1	GAGTCTGACGGTCCTCCTTG CAGCACAGCATCATGGTCCACATTA
Sb-dmc1 R1	AGTAACAGAGGATCAAGTTGTTGAAGAGG
Sb-nanog F1	CAACAAGGTTACCATATCATCATGCTCAC
Sb-nanog R1	CTAAGGAGGCACCAGAAGGATACCA
Sb-cyp19a F1	CCTGCTCCATATCTCTTCTCTTCTGTTG
Sb-cyp19a R1	GCTTGTGACCTGGCGATGACTC
Sb-β-actin-F1	CGGAATCCACGAGACCACCTAC
Sb-β-actin-R1	ACTCCTGCTTGCTGATCCACAT

## Data Availability

All data have been included in the manuscript.

## References

[1] Kuznetsova I.S., Thevasagayam N.M., Sridatta P.S.R., Komissarov A.S., Saju J.M., Ngoh S.Y., Jiang J., Shen X., Orban L. (2014). Primary Analysis of Repeat Elements of the Asian Seabass (*Lates calcarifer*) Transcriptome and Genome. Front. Genet..

[2] Yue G.H. (2025). Asian Seabass (*Lates calcarifer*): A Versatile Species for Marine and Freshwater Aquaculture. Rev. Aquac..

[3] Moore R. (1979). Natural Sex Inversion in the Giant Perch (*Lates calcarifer*). Aust. J. Mar. Freshw. Res..

[4] Guiguen Y., Fostier A., Piferrer F., Chang C.-F. (2010). Ovarian Aromatase and Estrogens: A Pivotal Role for Gonadal Sex Differentiation and Sex Change in Fish. Gen. Comp. Endocrinol..

[5] Baroiller J.F., D’Cotta H., Saillant E. (2009). Environmental Effects on Fish Sex Determination and Differentiation. Sex. Dev..

[6] Lehmann R. (2012). Germline Stem Cells: Origin and Destiny. Cell Stem Cell.

[7] Zuckerman S. (1951). The Number of Oocytes in the Mature Ovary. Recent Prog. Horm. Res..

[8] Bukovsky A., Gupta S.K., Virant-Klun I., Upadhyaya N.B., Copas P., Van Meter S.E., Svetlikova M., Ayala M.E., Dominguez R. (2008). Study origin of germ cells and formation of new primary follicles in adult human and rat ovaries. Methods Mol. Biol..

[9] Zou K., Yuan Z., Yang Z., Luo H., Sun K., Zhou L., Xiang J., Shi L., Yu Q., Zhang Y. (2009). Production of Offspring from a Germline Stem Cell Line Derived from Neonatal Ovaries. Nat. Cell Biol..

[10] White Y.A.R., Woods D.C., Takai Y., Ishihara O., Seki H., Tilly J.L. (2012). Oocyte Formation by Mitotically Active Germ Cells Purified from Ovaries of Reproductive-Age Women. Nat. Med..

[11] Xu H., Zhu X., Li W., Tang Z., Zhao Y., Wu X. (2018). Isolation and in Vitro Culture of Ovarian Stem Cells in Chinese Soft-Shell Turtle (*Pelodiscus sinensis*). J. Cell. Biochem..

[12] Liu X., Liu F., Xu H., Yang Y., Wang Y., Hong X., Li W., Yu L., Chen C., Xu H. (2022). Characterization of the In Vitro Cultured Ovarian Cells in the Asian Yellow Pond Turtle (*Mauremys mutica*). Biology.

[13] Wong T.-T., Saito T., Crodian J., Collodi P. (2011). Zebrafish Germline Chimeras Produced by Transplantation of Ovarian Germ Cells into Sterile Host Larvae. Biol. Reprod..

[14] Hong Y.H., Liu T.M., Zhao H.B., Xu H.Y., Wang W.J., Liu R., Chen T.S., Deng J.R., Gui J.F. (2004). Establishment of a Normal Medakafish Spermatogonial Cell Line Capable of Sperm Production in Vitro. Proc. Natl. Acad. Sci. USA.

[15] Okutsu T., Shikina S., Kanno M., Takeuchi Y., Yoshizaki G. (2007). Production of Trout Offspring from Triploid Salmon Parents. Science.

[16] Lee S., Katayama N., Yoshizaki G. (2016). Generation of Juvenile Rainbow Trout Derived from Cryopreserved Whole Ovaries by Intraperitoneal Transplantation of Ovarian Germ Cells. Biochem. Biophys. Res. Commun..

[17] Lakra W.S., Swaminathan T.R., Joy K.P. (2011). Development, Characterization, Conservation and Storage of Fish Cell Lines: A Review. Fish Physiol. Biochem..

[18] Zhong C., Tao Y., Liu M., Wu X., Yang Y., Wang T., Meng Z., Xu H., Liu X. (2022). Establishment of a Spermatogonial Stem Cell Line with Potential of Meiosis in a Hermaphroditic Fish, *Epinephelus coioides*. Cells.

[19] Li W., Ding S. (2010). Small Molecules That Modulate Embryonic Stem Cell Fate and Somatic Cell Reprogramming. Trends Pharmacol. Sci..

[20] Kanatsu-Shinohara M., Ogonuki N., Inoue K., Miki H., Ogura A., Toyokuni S., Shinohara T. (2003). Long-Term Proliferation in Culture and Germline Transmission of Mouse Male Germline Stem Cells. Biol. Reprod..

[21] Hong Y.H., Winkler C., Schartl M. (1996). Pluripotency and Differentiation of Embryonic Stem Cell Lines from the Medakafish (*Oryzias latipes*). Mech. Dev..

[22] Goswami M., Yashwanth B.S., Trudeau V., Lakra W.S. (2022). Role and Relevance of Fish Cell Lines in Advanced in Vitro Research. Mol. Biol. Rep..

[23] Fan L.C., Crodian J., Collodi P. (2004). Culture of Embryonic Stem Cell Lines from Zebrafish. Methods Cell Biol..

[24] Fang F., Gong Z., Guo C., Wang C., Ding L., Zhou B., Chen S. (2025). Establishment of an Ovarian Cell Line from Tomato Grouper (*Cephalopholis sonnerati*) and Its Transcriptome Response to ISKNV Infection. Fish. Shellfish. Immunol..

[25] Sun A., Wang T.Z., Wang N., Liu X.F., Sha Z.X., Chen S.L. (2015). Establishment and Characterization of an Ovarian Cell Line from Half-Smooth Tongue Sole *Cynoglossus semilaevis*. J. Fish Biol..

[26] Yuan Y., Hong Y. (2017). Medaka Insulin-like Growth Factor-2 Supports Self-Renewal of the Embryonic Stem Cell Line and Blastomeres in Vitro. Sci. Rep..

[27] Choi J.H., Ryu J.H., Gong S.P. (2023). Establishment and Optimization of an Aggregate Culture System of Testicular Cells from Marine Medaka, *Oryzias dancena*. J. Mar. Sci. Eng..

[28] Watanabe A., Hatakeyama N., Yasuoka A., Onitake K. (1997). Distributions of Fibroblast Growth Factor and the mRNA for Its Receptor, MFR1, in the Developing Testis of the Medaka, *Oryzias latipes*. J. Exp. Zool..

[29] Adashev V.E.E., Kotov A.A.A., Olenina L.V.V. (2023). RNA Helicase Vasa as a Multifunctional Conservative Regulator of Gametogenesis in Eukaryotes. Curr. Issues Mol. Biol..

[30] Chambers I., Colby D., Robertson M., Nichols J., Lee S., Tweedie S., Smith A. (2003). Functional Expression Cloning of Nanog, a Pluripotency Sustaining Factor in Embryonic Stem Cells. Cell.

[31] Henderson J.K., Draper J.S., Baillie H.S., Fishel S., Thomson J.A., Moore H., Andrews P.W. (2002). Preimplantation Human Embryos and Embryonic Stem Cells Show Comparable Expression of Stage-Specific Embryonic Antigens. Stem Cells.

[32] Hou P., Li Y., Zhang X., Liu C., Guan J., Li H., Zhao T., Ye J., Yang W., Liu K. (2013). Pluripotent Stem Cells Induced from Mouse Somatic Cells by Small-Molecule Compounds. Science.

[33] Ying Q.-L., Wray J., Nichols J., Batlle-Morera L., Doble B., Woodgett J., Cohen P., Smith A. (2008). The Ground State of Embryonic Stem Cell Self-Renewal. Nature.

[34] Watanabe K., Ueno M., Kamiya D., Nishiyama A., Matsumura M., Wataya T., Takahashi J.B., Nishikawa S., Nishikawa S., Muguruma K. (2007). A ROCK Inhibitor Permits Survival of Dissociated Human Embryonic Stem Cells. Nat. Biotechnol..

[35] Li W., Wei W., Zhu S., Zhu J., Shi Y., Lin T., Hao E., Hayek A., Deng H., Ding S. (2009). Generation of Rat and Human Induced Pluripotent Stem Cells by Combining Genetic Reprogramming and Chemical Inhibitors. Cell Stem Cell.

[36] Yi M., Hong N., Hong Y. (2010). Derivation and Characterization of Haploid Embryonic Stem Cell Cultures in Medaka Fish. Nat. Protoc..

[37] Yi M., Hong N., Hong Y. (2009). Generation of Medaka Fish Haploid Embryonic Stem Cells. Science.

[38] Xu H.-Y., Hong X.-Y., Zhong C.-Y., Wu X.-L., Zhu X.-P. (2022). Restoring Genetic Resource through In Vitro Culturing Testicular Cells from the Cryo-Preserved Tissue of the American Shad (*Alosa sapidissima*). Biology.

[39] Yao J., Zeng L., Zhu Z., Feng K., Zhou C., Wang W., Zhou J., Su S., Xu H. (2025). Studies on Ovarian Tissues’ Cryopreservation in the Cyprinid Species. Cryobiology.

